# Enhancing translation capacities of COVID-19 researchers through interdisciplinary collaboration: empathy, awareness, and action

**DOI:** 10.1080/17482631.2026.2666722

**Published:** 2026-05-01

**Authors:** Lahari Yaddanapudi, Erik Fisher, Julia Hahn

**Affiliations:** a Institute for Technology Assessment and Systems Analysis, Karlsruhe Institute of Technology, Karlsruhe, Baden-Württemberg, Germany; b School for the Future of Innovation in Society, College of Global Futures, Arizona State University, Tempe, Arizona, USA

**Keywords:** Knowledge translation, socio-technical integration, health crises governance, capacity building, reflexivity, public trust

## Abstract

**Purpose:**

Integrating societal considerations into public health research, particularly during crises, can foster public trust, support inclusive policy-making, and enhance technology development. Interdisciplinary collaboration may enhance researchers' capacities to reflect on the societal impacts of scientific advancements as they occur.

**Methods:**

This qualitative Socio-Technical Integration Research (STIR) study investigated these claims among four early-career researchers in virology, physics, and engineering, working in a large-scale COVID-19 research project across Germany. The collaboration involved 12 weeks of protocol-based dialogue exercises, pre- and post-study interviews, and participant observation, and was analyzed using the “Midstream modulation” framework.

**Results:**

We found that the exercises documented, and in some cases stimulated, changes in participants' awareness, attitudes, and behaviours regarding their research's broader social context. One participant grew more aware of their work's social impact over time, recognizing stakeholders beyond the laboratory. Another shifted attitudes toward science communication, while a third demonstrated greater empathy for public reactions to scientific advice.

**Discussion:**

These enhanced capacities for reflexivity suggests potential of STIR for improved communication among scientists, the public, and policymakers, strengthening the science–society interface in COVID-19 and broader health research. Such collaborations can build public trust, inform interventions, and improve the translation of basic research into effective health policies.

## 1. Background

Interdisciplinary collaboration is widely recognized as a key driver of innovation and societal relevance in health research. Studies have shown that teams integrating diverse disciplines—from epidemiology and clinical science to social sciences, ethics, and public policy—produce research that is more applicable to real-world contexts and more responsive to public concerns (Crocker et al., 2018; Domecq et al., 2014; Greenhalgh et al., 2019). Such collaborations are particularly crucial during health crises, where decision-making must contend with uncertainty, rapidly changing scientific knowledge, and urgent societal needs. The COVID-19 pandemic, for example, highlighted both the potential and the shortcomings of health research systems in mobilizing evidence to guide effective public policy (Glover et al., 2020; Greenhalgh et al., 2020).

A growing interdisciplinary literature has also examined how the pandemic disrupted research practices themselves, drawing attention to challenges such as rapidly shifting evidentiary standards, constraints on data collection, pressure to produce actionable knowledge under uncertainty, especially in health and clinical research where the urgency of the pandemic was most acute (Bramstedt, 2020; Hlatshwako et al., 2021; Keen et al., 2022; Kosciejew, 2022; Mourad et al., 2020; Newman et al., 2021; Sohrabi et al., 2021; Sy et al., 2020). This highlights how health emergencies expose the fragility of research infrastructures and affect research areas differently, with fields directly linked to pandemic response—like virology, epidemiology, and public health—experiencing especially intense pressures (Becker, 2024). While these studies primarily analyze research systems and crisis conditions at a macro level, they underscore the importance of understanding how individual researchers navigate uncertainty, responsibility, and translation in practice. Our study complements this literature by focusing on the micro-level capacities through which scientists reflect on and adapt their research practices in situ.

During crises, the stakes of evidence-informed policy are also heightened. Public health emergencies demand timely action, yet the evidence base is often incomplete or evolving. In such situations, gaps frequently emerge between scientific research and policy implementation. Reflexivity—defined as awareness of how one's thoughts and activities interact with one's social context—can enhance the capacity of researchers to anticipate and critically engage with the broader societal and ethical implications of their work, helping to bridge this gap. It allows scientists to identify potential barriers to effective communication and implementation and to adjust their approaches accordingly (Owen et al., 2012; Stilgoe et al., 2013). Engaging in “reflective practice” in the context of healthcare and health systems is thought to bridge the gap between theory and practice, especially in complex or uncertain situations, and makes professionals more attuned to the needs of patients, teams, and communities (Salter & Kothari, 2016). Reflexivity, therefore, becomes a vital component of responsible science, particularly when societal trust and swift, coordinated responses are essential.

Frameworks like Evidence-Based Practice (EBP), Evidence-Informed Policymaking (EIP), Knowledge Translation (KT), and Integrated Knowledge Translation (IKT) have sought to institutionalize connections between researchers, policymakers, and practitioners, albeit often emphasizing system-level mechanisms. These approaches emphasize the co-production of knowledge, ensuring that scientific evidence is not only produced but also mobilized effectively to shape policies that are practical, equitable, and aligned with societal needs (Kothari & Wathen, 2013; Lavis et al., 2009). However, this literature has tended to focus on institutional arrangements, governance frameworks, and collective mechanisms that enable such co-production, while empirical attention to the role of individual scientists in these processes remains limited. By contrast, reflective practice methods highlight that knowledge is shaped by personal experience and specific contexts (Salter & Kothari, 2016). One such method, Socio-Technical Integration Research (STIR), introduces structured reflexive dialogues within research environments, enabling scientists to systematically consider the societal implications of their decisions (Smolka et al., 2021). Across the broader literature on knowledge co-production and science–policy engagement, reflexivity is largely located within institutional mechanisms and collective processes, leaving the micro-level practices through which individual scientists develop and exercise reflexive capacities underexplored. STIR offers a methodological advantage in this regard by creating structured opportunities to examine reflexivity as an individual, situated practice within everyday research settings, as its decision protocol systematically prompts researchers to identify and reflect on value considerations embedded in routine decisions. (Smolka & Fisher, 2024). This approach has been applied in numerous fields from nanotechnology to artificial intelligence, yet remains underutilized in health research. In this study, we used the STIR method to explore how interdisciplinary dialogue can support, and if possible, enhance individual capacities for reflexivity and provide empirical evidence to assess whether such exercises can enhance reflexive, inclusive, and socially responsive health research and scientific practices.

The objective of this paper, therefore, is to examine the micro-level capacities of individual scientists through the STIR method of interdisciplinary collaboration. It seeks to provide evidence of reflexivity that is cultivated through structured engagement and how reflexivity contributes to the broader goals of socially responsive, actionable health research. It represents a form of research on research—an investigation into how research is done, how it interacts with society, and how it can be improved to better support public health. In doing so, it offers insights into how more responsive and integrative scientific practices can be fostered—not only during emergencies, but as a standard part of health research ecosystems.

## 2. Methods

### 2.1. Data collection

**Figure 1.**
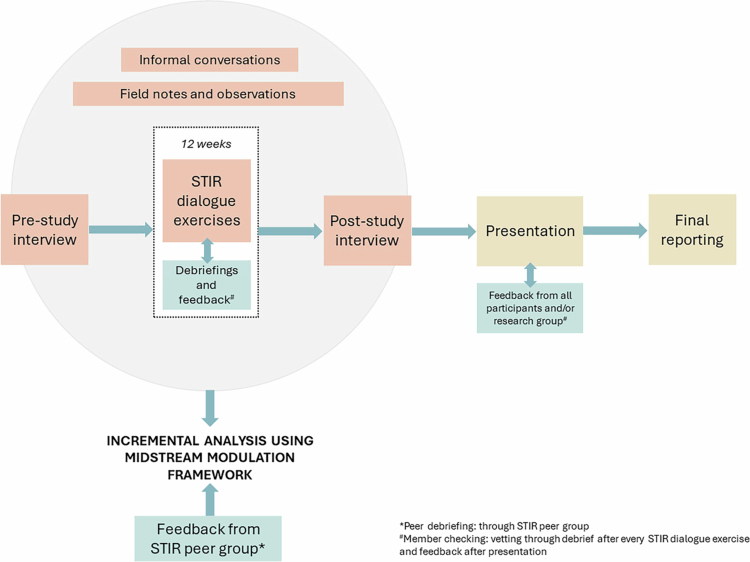
The STIR process.

The data for this study was collected according to the STIR method that includes a semi-structured pre-study interview, 12 weekly decision protocol exercises, and a post-study interview (Figure 1). The study included five participants who were recruited selectively to achieve comparability across and variability within this large-scale project. The selection criteria were: same rank (early career researchers^a^), similar responsibilities (reporting, research, science communication, and light administration), involvement in interdisciplinary exchange, approximately the same start time (in the project), representation from all three work packages of the project, and a gender balance. All of these criteria allowed for a robust study in the project's context, and a sample size of five is fairly typical for a STIR study. One participant dropped out of the study after 5 weeks (due to time constraints), hence data from that participant were not included in this study.

The pre- and post-study interviews consist of identical questions, with additional follow-up questions in the post-study interview. STIR dialogues are semi-structured exercises that represent the main procedural component and that follow a 2 × 2 grid containing:

- Opportunity: a decision that the participant needs to make in the near future
- Considerations: factors that need to be considered when making the decision
- Alternatives: different ways to address the opportunity
- Outcomes: the most probable alternative(s) and its potential consequences

The study is carried out by an “embedded humanist”—typically, a social science or an STS researcher, but in this case, a qualitative public health researcher—who guides the participant through the decision protocol exercise using questions, going through each of the quadrants and revisiting them as needed. Importantly, it is the STIR protocol itself, rather than the humanist's presence, that prevents the introduction of values, preferences, suggestions, and other potential influences. The protocol employs Socratic-style questioning that prompts participants to reflect on their own values and societal considerations (Poznic & Fisher, 2021; Smolka & Fisher, 2024). The embedded humanist tailors their questions to draw out these reflections and societal considerations, without imposing interpretations. Each exercise ends with “member checking” that takes the form of a debrief during which participants are invited to vet the embedded humanist's data and interpretations.

This study followed a hybrid format. The first session for all participants took place in person, during which the embedded humanist also toured their work environments, as an integral part of the STIR method involves “embedding” of the social scientist into the participant's environment. These included chemistry laboratories, biosafety levels 2 and 3 laboratories, engineering labs, and office spaces, which provided contextual insights into their research settings. Following this initial visit, two participants continued with in-person STIR sessions, while the remaining three engaged in virtual sessions, due to them being geographically spread throughout Germany. For online participants, observational data was limited to expressions, tone of voice, mood, and body language during the discussions, while in-person sessions allowed for a more immersive understanding of participants' work environments and interactions.

Although an ethics approval was deemed non-obligatory by the institutional ethics committee, we ensured the following to align our study with international norms of ethical transparency in qualitative research, including principles from the Declaration of Helsinki. All participants provided their verbal informed consent (on record) for voluntary participation in the study, and were allowed to withdraw their consent at any point. This approach was chosen to protect participant anonymity and reduce data linkage risks, while minimizing procedural intrusion in a conversational setting. The consent process was audio-recorded, and participants were provided with all study information in advance. The study recordings and other related data were stored in compliance with the GDPR. Any identifiers, including gender and field of specialization (and all the content that is directly attributable to participants), have been carefully screened for and removed. The participants read the draft of this article and provided written informed consent for publication.

### 2.2. Data analysis

All the data from the interviews, decision-protocol exercises, field observations, and notes were analyzed using the analytical lens of Midstream Modulation (MM) framework; during the study (incremental analysis) and after. The term “midstream” refers to the research and development phase of innovation, while “modulations” are the gradual changes resulting from decisions that subtly influence the direction of research and innovation. Together, midstream modulation describes these socio-material shifts as they emerge through scientists' and engineers' reflective engagement with broader societal contexts. These changes can unfold in a modulation sequence—starting as unreflective (de facto), becoming conscious (reflexive), and eventually evolving into intentional (deliberate) actions (Fisher et al., 2006).

**De facto modulation** occurs during a STIR dialogue when contextual factors—such as pre-existing problem definitions, implicit values, emotional undercurrents, or assumptions about society, the public, and ethics—subtly influence scientific or technical practices without being explicitly recognized. In contrast, **reflexive modulation** takes place when the researcher becomes aware of these underlying influences and recognizes their impact on the research process, opening the possibility for intentional change. Following reflexive modulation, when a researcher consciously recognizes the influence of contextual factors, a tangible change in practice may be implemented. This observable shift is referred to as **deliberate modulation**.

Rather than using conventional inductive or deductive coding, STIR employs an incremental analysis approach in which the embedded humanists use these three modulation phases to tag and organize data, and document potential indications of modulation sequences, often while such sequences are still unfolding. This allows continuous tracing of participants' reflections and shifts in practice over the course of the study. Modulation sequences (Table II) were derived from this longitudinal documentation across sessions, supported by field notes, memos, and debriefings, rather than retrospective coding of transcripts. To increase accuracy and credibility, embedded humanists use triangulation among exercise and interview transcripts, field notes and observations, and “member checking” with participants and their peers. STIR allows for incremental MM analysis to influence the questions embedded humanists ask during exercises and debriefings, provided questions do not introduce or encourage specific values, preferences, or suggestions. To minimize such introductions, embedded humanists document their methodological choices using field notes and reflexive memos. Methodological choices are also scrutinized by members of the STIR community during peer debriefings with mentors and peer group as well as through member checking with research participants. For instance, the embedded humanist in this study presented her initial findings to participants in poster presentations during a PhD school as well as in formal presentations that included all participants, in both cases, encouraging participant feedback.

In this paper, we first present the results of changes observed as a comparison of responses of the participants for the same question in the pre- and post-study interviews, and then present the analysis through the MM framework.

## 3. Results

We identified changes across three dimensions—awareness, attitudes, and behaviours—emerging at three distinct yet interrelated levels: (1) the connection between science and society, (2) recognition of the unintended consequences of one's own research, and (3) shifts in individual research practices (see Table I). While the three dimensions are explained in detail in later parts of the paper, the three levels were derived based on inductive coding of the modulation sequences. “Science-society connection” represents all discourse within which the relationship between science, research, and technology development was discussed in relation to society, in both directions. “Unintended consequences of research” includes discourse regarding the consequences of scientific research. “Individual research practices” refers to the participants' day-to-day practices and the values that govern them. In this paper, we present findings related to the first level: the science–society connection.

**Table I.** Key results of the STIR study.

	Change in awareness	Change in attitude	Change in behaviour
**Science-society connection**	*[P1]* Money matters	*[P3]* It's not just about data (3.2.1.)	*[P4]* “From trade-off to synergy” modulation sequence (3.3.1.)
	*[P2]* Beyond the laboratory walls (3.1.1.)	*[P4]* From trade-off to synergy (3.2.2.)	
	*[P2]* Who counts as a stakeholder? (3.1.2.)		
**Unintended consequences of research**	*[P2]* Collective environmental impact^a^		
**Individual research practices**	*[P2]* Collective scientific good^^a^^	*[P2]* Their own role/responsibility	*[P1]* Money matters
			*[P2]* Collective scientific good^^a^^

^a^ These three vignettes are discussed in detail in a forthcoming book by Fisher et al., which includes the full modulation sequence as well as a comprehensive narrative tracing the participant's incremental changes over time.

### 3.1. Change in awareness

The concept of social context is a key principle for understanding how scientific knowledge and technological innovations are not only products of but also influence and are influenced by their environments. This context impacts, often profoundly, the actions, perceptions, and feelings of individuals within it. By examining the social context, researchers can discern the multifaceted interactions between individuals and the broader social, cultural, and institutional settings that shape and are shaped by their actions (Coghlan & Brydon-Miller, 2014; Funtowicz & Ravetz, 1993; Gibbons et al., 2010). From this awareness of the social context, more collective goal-oriented modulations can possibly be developed (Fisher, 2019).

Across all participants, the most common change in awareness was about who they considered as stakeholders of their study. While P1 recognized the role of the public in determining scientific funding, P2 broadened views on the consequences of their research on the scientific community as well as the end-users of their technology; and included society as a relevant stakeholder, other than only the project partners and companies they collaborate with.

To illustrate this change in the STIR study participants, let us consider the differences in the pre-study and post-study interview of P2. In both the interviews, the same questions were asked, yet significant changes were observed in P2's answers.

#### 3.1.1. Beyond the laboratory walls

In the pre-study interview, when P2 was asked what they thought the social aspects of their work were, their response reflected an immediate and narrowly defined understanding of social context:

*"…the social aspects of this are more like questions in the direction of, for example, the **security of our facility**...” “…When talking to friends or family, I'm trying to put as much motivation into explaining what I'm doing as possible because I think it's really important to **keep people interested**...”* [P2, pre-study interview]

This perspective indicated limited social awareness and was focused on the immediate infrastructure necessary for P2's activities. Moreover, when discussing communication with friends and family, the participant revealed an attempt to generate interest and maintain engagement within their personal network. However, the underlying motivation appeared to be the preservation of personal interest and support, rather than a broader societal impact.

In contrast, the post-study interview revealed a marked shift in the participant's awareness and articulation of social context when asked the same question, showing a more nuanced understanding of impact on not just scientists and collaborators, but also the broader society:

*"…I'm trying to always find a way or always put the project in a way so that it could really benefit some **real-life consequences**. That is the impact, I think.” “The best thing I can do is try to do research that **benefits society** in some way in the end. That would be then my consideration of giving something back of the money that I get from society.”* [P2, post-study interview]

This highlighted an increased awareness of the broader societal implications of the participant's work. The focus expanded from a limited concern to a broader societal impact, indicating a significant shift in the participant's understanding of their work's relevance and potential benefits. The participant's motivation shifted from generating immediate interest among friends and family to a more profound commitment to societal benefit. Additionally, the participant also actively acknowledged that their work was supported by public funding, further deepening the purpose and responsibility of that work. These changes reflect a deeper engagement with the social context, where the participant saw their scientific work as part of a larger societal fabric and strived to contribute meaningfully to it.

#### 3.1.2. Who counts as a stakeholder?

Similar changes in the awareness of social context were reflected in the answers given by all participants regarding who they regarded as stakeholders in their project. These shifts show in the post-study interview and imply a better understanding of who the stakeholders are, including coming to understand the public as stakeholders. For example, in the pre-study interview, participant P2's identification of stakeholders was limited to project partners and companies they collaborate with, indicating a narrow, project-centric view of engagement. This perspective suggested that the participant primarily considered immediate, direct collaborators as the primary stakeholders, without necessarily recognizing the broader societal implications or potential beneficiaries of their work. However, in the post-study interview, this participant's understanding of stakeholders broadened significantly, encompassing not only direct commercial stakeholders but also the general public, highlighting a deeper awareness of the societal impact and a recognition of the public as a crucial stakeholder group:

*"It would be companies that produce air purification systems. It could be transport companies like airlines, or public transport, companies that could benefit. In the end, it could be **people in public spaces, just any kind of people in public spaces**. If it would be possible to make public spaces **more safe** with the help of our research”* [P2, post-study interview]

The participant's acknowledgement of the diverse range of potential beneficiaries underscored an evolved understanding of the interconnectedness between their research and the wider social environment.

This transformation—from seeing social context as an internal concern to understanding its societal implications—is crucial. It reflects a deepening of scientific responsibility, where research aims are no longer understood mainly as technical advancements or industry applications, but about how these developments can serve society. The participant's growing awareness of stakeholders and social impact is an indicator of a tangible change in perspective. It shows that, through reflective engagement during the STIR study, they have moved from a localized, infrastructure-focused understanding to a broader, more inclusive view of their work's significance.

By the end of the study, social context was no longer just about securing a facility or explaining research to friends for P2 and other participants. It had become about real-world consequences, ethical considerations, and ensuring that science serves the public in meaningful ways. This kind of shift is crucial for fostering socially responsible research—where scientists are not just experts in their fields but also actively aware of and engaged with the societies they impact.

### 3.2. Change in attitude

Attitude change can be induced through thoughtful processes, where an individual carefully considers and evaluates persuasive arguments, or through less effortful means, such as associating the attitude object with positive emotions or relying on simple cues like the source's credibility. Changes resulting from thoughtful consideration are usually more enduring, resistant to counterarguments, and better predictors of future behaviour (Petty, 2001). In our study, participants demonstrated significant changes in attitude towards societal considerations, as observed from the answers to the same question in the pre- and post-study interviews.

#### 3.2.1. It's not just about data

Comparing the pre-study and post-study interview responses of participant P3 to the same question suggests a notable change in attitude towards societal considerations. In the pre-study interview, in their response to the interviewer's question of the role of scientists in making research better, P3 recognized the need for better science communication, focusing on the technical aspect of making data more accessible and understandable for non-scientists:

*"We need to make our data **better and available to understand for non-scientists**, because it's difficult to read a paper, understand how the paper is written, and how to get the key value out of the paper.”* [P3, pre-study interview]

This perspective was largely concerned with the format of disseminating the outcomes of research to a wider audience. The emphasis was on the difficulty that non-experts face in extracting key values and understanding the structure of scientific literature. The underlying assumption here was that alternative ways of data sharing other than through scientific papers would lead to better understanding among the general public.

By comparison, in the post-study interview, the participant's response to the same question shifted significantly in two ways –responding with heightened empathy, as well as taking more responsibility, suggesting a shift in attitude:

*"…there were all these different groups of people that didn't understand what was going on and they got **scared and frustrated**, and then there was **anger** and everything. It's **very important to communicate science**, researchers and scientists all over the world, **we need to do our part in that**.”* [P3, post-study interview]

The participant then emphasized the emotional responses of the public—fear, frustration, and anger—highlighting the importance of effective science communication to mitigate these feelings. Shifting from alternative data-sharing approaches, the participant now demonstrates empathetic engagement with the public's emotional experiences and takes responsibility for fostering effective science communication. This change in attitude is significant as it demonstrates a deeper engagement with the societal impact of scientific work. This broader view acknowledged the emotional and psychological impact of scientific communication on the public, highlighting the importance of empathy and effective communication strategies to alleviate these negative emotions. This is a meaningful change—useful for fostering public trust in science and supporting more inclusive, effective public engagement—because it enhances the relevance and effectiveness of science communication, ensuring that scientific knowledge is not only accessible but also comprehensible and reassuring to the public. Such a reflection suggests that the participant is more aware of the possibility of “sociological ambivalence”, which has been associated with public controversies and distrust of scientific expertise (Beck, 1992; Giddens, 1991; Irwin & Michael, 2003). Moreover, the participant's statement of the need to do their part displays a motivation to take greater responsibility in addressing the roots of such distrust. Taking greater responsibility often requires a shift from a detached to a more personally relevant attitude of empathy towards the public's emotional responses, as we see in this example. Attitudinal changes that acknowledge and engage with public concerns can foster a more inclusive approach and lead to more effective science communication strategies that foster greater public understanding and trust.

#### 3.2.2. From trade-off to synergy

A change in attitude was also demonstrated in participant P4's pre- and post-study interviews about the value of science communication and the trade-off with its impact on their scientific efforts. In the pre-study interview, this participant acknowledged that public engagement makes their work meaningful but views it as an additional burden that detracts from their primary research efforts.:

“…*my energy is limited. **If I need to show my results to the public, I need to decrease my time in the lab**. However, the good thing is again, the public could know more details about your work. That makes my work meaningful, I think.”* [P4, pre-study interview]

There is an internal conflict in this statement. On the one hand, the participant acknowledged the importance of sharing research with the public, but on the other, they framed it as a trade-off—a necessary but burdensome task that takes away from real scientific progress. Their words reflected reluctance, as though public engagement is an obligation rather than an integral part of scientific work. While they saw its purpose, they remained primarily concerned with the cost to their own research efforts.

However, by the post-study interview, the participant's attitude had transformed significantly. They see public feedback as a synergistic source of motivation and encouragement.

*"…I got very nice reflections from the public. This is going to **encourage me** to move forward, to study more things about this topic. Wow.” “I could be **more confident** in doing scientific research, especially on this topic… ...because then I'm going to feel like I'm on a good trajectory. I'm doing something really good for the public. This is going to **encourage me and make me confident** in doing such things.”* [P4, post-study interview]

The participant described public feedback as influential and empowering, boosting the participant's confidence and enthusiasm for their research. This shift from viewing public engagement as a burden for science to seeing it as a beneficial and motivating factor for science demonstrated a substantial change in attitude. The participant was no longer hesitant about communicating science; they saw it as fuel for their work, reinforcing that they were on the right track. The participant's exclamation “Wow” pointed to their surprise at this unexpected development—science communication is not just about informing the public, it can also support the mutual benefit of scientists and society.

Shifts in attitude, such as we have just considered, are crucial not only for individual researchers, but more broadly for public trust in science. Scientists who feel disconnected from society may struggle to effectively communicate the importance of their work. However, when researchers find motivation in public engagement, it creates a more open and reciprocal relationship between scientists and their public interlocutors. Such shifts can help bridge the gap between scientific research and public understanding, fostering stronger trust, dialogue, and shared investment in scientific progress.

Importantly, such shifts in attitude are also more likely to be sustainable when they emerge from thoughtful consideration (Crano & Gardikiotis, 2015; Petty, 2001). In each of the examples, thoughtful consideration was encouraged by the reflective practices of STIR dialogues, which often led to changes in action, which we illustrate next.

### 3.3. Change in behaviour

A widely accepted understanding of behaviour change is that it unfolds gradually over time, rather than occurring as a single, definitive event. Change often progresses through stages, with individuals moving from intention to action in an incremental way. Just as significant lifestyle changes—like quitting smoking or altering one's diet—rarely happen overnight, shifts in professional behaviour, such as changes in research practice or stakeholder engagement, tend to emerge progressively (Glanz, 2025). In our study, participants' reflexive insights often translated into a stated intention to act differently or subtle shifts in how they approached their work—signalling early steps in a broader behavioural change process (Prochaska et al., 2015).

#### 3.3.1. “From trade-off to synergy” modulation sequence

The change in attitude presented in Section 2.2 emerged from a series of events in which the participant reflects on their engagement with the public during interviews and STIR dialogues. The modulation sequence (Table II) offers a clear trajectory of the participant's evolving relationship with science communication, effectively illustrating how their stance shifted from initial resistance, to reflexive awareness, to deliberate commitment. The midstream modulation framework, which captures how actors reflect on and potentially reorient their socio-technical practices during the course of ongoing activity, is particularly useful in tracing this participant's transformation across time.

**Table II.** “From trade-off to synergy” modulation sequence.

Nr.	Week	Modulation Phase	Description
1	Week 1	De-facto	Science communication and public engagement will interfere with P4's research and “time in the lab”
2	Week 2	Reflexive	Participant 4 emphasizes the value of “being confident” in their results, explaining that this confidence is important for “people to trust your result,” and notes that one way to achieve this is by “having conversations with the public.”
3	Week 2	Reflexive	Participant recognizes the value of sharing results to the public: “people could follow my suggestions (about prevention of COVID)” and that “scientific research is not just for yourself, it is for the world”
4	Week 12	Reflexive	After getting “very nice reflections from the public,” participant feels ***encouraged*** and ***confident*** “to move forward, to study more things about this topic. Wow”.
5	Week 12	Deliberate	“I [will] absolutely join the [institute's open science day] and would like to show my results and to design something to transfer the knowledge to the public.”

##### 3.3.1.1. De-facto modulation: science communication as a burden

In the pre-study interview, which took place during the first week of the study, the participant expressed a default, unexamined stance towards public engagement: that it inherently competes with the core demands of scientific research (Nr.1, Table II). This de-facto modulation starting point reveals an internalized norm, for the participant, but also within scientific culture—that public communication, while perhaps valuable in theory, is practically disruptive or even detrimental to a scientist's main responsibilities. The participant's framing of engagement as interference suggested a compartmentalized view of science and society, where the former is pursued in isolation from the latter.

##### 3.3.1.2. Reflexive modulation: public engagement as trust-building

By week 2, during a STIR dialogue that consisted of an in-the-moment discussion of a research decision, the participant began to reflect more actively on the role of the public in the scientific process. Recognizing that confidence in one's results is tied to public trust, and that engaging with the public might foster such trust, signalled a significant move toward reflexive modulation (Nr. 2, Table II). The participant was no longer simply stating an inherited view but was questioning and reconstructing the rationale behind why public engagement might be relevant—not as an obligation, but as part of doing good science.

The reflexivity deepened when the participant articulated a social and ethical rationale for sharing their results, especially in the context of COVID-19. As the quotes (Nr. 3, Table II) show, a re-evaluation of the purpose of science itself, aligning it with societal benefit, took place. This shift reflects the kind of value-based reasoning encouraged by STIR dialogues—where technical work is understood not in isolation, but in relation to broader social goals.

##### 3.3.1.3. Deliberate modulation: from reflection to action

By week 12, the participant's stance moved from reflective insight to a concrete change in disposition and future intent, representing a transition from reflexive to deliberate modulation. Describing feedback from the public as emotionally and intellectually affirming (Nr. 4, Table II), the participant linked engagement not only to social responsibility but also to personal and professional motivation. This feedback loop—where engagement fuels scientific enthusiasm—contrasts starkly with the initial view of engagement as a burdensome trade-off.

The final resolve (Nr. 5, Table II) pointed to a deliberate modulation: the participant is not only supportive of engagement in theory but was now planning specific actions to institutionalize it within their scientific practice. This demonstrates a fully reoriented perspective—science communication had transitioned from a peripheral and costly trade-off to an integral and generative synergistic activity.

This modulation sequence is a vivid example of how midstream modulation enables shifts in scientific practice not through external mandates, but through internal reflection and evolving sense-making. The participant moves from a default, somewhat dismissive stance towards public engagement, through layers of reflection that tie engagement to trust, social impact, and personal growth, culminating in deliberate intent to continue such practices. The change is not just attitudinal but practical and normative, suggesting that structured opportunities for reflection—such as those offered in midstream modulation—can help researchers reframe science communication not as a distraction from research, but as a dimension of responsible and meaningful science itself.

Other participants, P1, and P2, also had concrete behavioural shifts. P1 worked towards finding new ways to make their product more cost-effective, recognizing that cost-benefit is a major concern for access to and acceptability of such technologies. P2, upon reflecting on their own role in managing the project funding, realized that they can take up more responsibility, and proactively initiated redistribution of project funding within their own research group. They also resolved to plan it better for the next project they get—exemplifying another case where a change in awareness and attitude lead to a change in behaviour. Together, these examples illustrate enhanced translation capacities, understood here as increased reflexivity, responsiveness to stakeholder needs, and the ability to anticipate and adapt research practices in relation to broader societal contexts.

## 4. Discussion

### 4.1. The role of interdisciplinary collaborations in enhancing the societal relevance of health research

Our findings contribute to a growing body of evidence on the role of interdisciplinary collaboration in enhancing the societal relevance of health research. The structured dialogue facilitated by the STIR method helped researchers reflect on how disciplinary boundaries shape their assumptions, and encouraged critical consideration of social, ethical, and public policy dimensions of their work, particularly by shifts in awareness, attitude, and behaviour towards these considerations. Empirically, this reflection became visible when participants reevaluated or rearticulated their understanding of the social impact of their micro-level practices, indicating that these assumptions were not only recognized but actively renegotiated during the sessions. The results underscore previous studies that show interdisciplinary research can yield more relevant, actionable knowledge (Greenhalgh et al., 2019; Klein, 2008; Oliver et al., 2019).

This is especially important during health crises, where scientific knowledge is rapidly evolving and must be integrated with societal needs and contexts in real-time (Yin et al., 2021). The efficacy of the interdisciplinary interactions analyzed here stems, in part, from the creation of structured spaces where researchers can engage in midstream reflection on the societal implications of their work. As prior studies have shown, such structured dialogues over time can foster trust, mutual understanding, and the surfacing of implicit assumptions that may otherwise remain unexamined (Flipse & Van De Loo, 2018; Lukovics & Fisher, 2017; Schuurbiers, 2011; Smolka et al., 2021). In the context of public health crisis, where evidence must often be mobilized rapidly and equitably, such qualities are crucial. During the COVID-19 pandemic, delays and misalignments between evidence generation and policy implementation hindered effective public health responses, underscoring the need for mechanisms to align research, data interpretation, and decision-making processes for rapid and context-sensitive translation (El-Jardali et al., 2020). In our study, this mechanism materialized through structured interdisciplinary dialogues that support reflexivity during the midstream of scientific practice. Interdisciplinary research on the pandemic has documented how such crises disrupt research infrastructures, evidentiary standards, and the conditions under which knowledge is produced and mobilized (Keen et al., 2022; Mourad et al., 2020; Newman et al., 2021; Sohrabi et al., 2021; Sy et al., 2020). While this work primarily examines research systems at a macro level, our findings complement these analyses by showing how individual researchers develop enhanced awareness and changed attitudes towards navigating these challenges through structured reflexive dialogue. Participants' engagement with translation issues strengthened anticipatory capacity of researchers, evidenced by participants' enhanced awareness of “real-life consequences” and forming a more empathetic view of public concerns, fostering greater preparedness for evidence-based policy engagement under uncertainty.

Importantly, the STIR method enhanced participants' translation capacities by changing how they approached research decisions. For example, all participants reported willingness to participate in science-communication activities, reconsidering assumptions about how it might be a trade-off with their research time. Similarly, two participants revised their ideas about using project funding and the importance of efficient distribution, and actively adjusting their behaviour to achieve it. This aligns with the principles of responsible innovation, which call for the inclusion of reflexivity, anticipation, responsiveness, and inclusion throughout the research process (Owen et al., 2012; Stilgoe et al., 2013). By applying these principles at the level of individual practice, our study demonstrates how interdisciplinary collaboration can act as a conduit for broader societal engagement and value-sensitive research design, rather than as a post-hoc addition to scientific inquiry.

By fostering these shifts in how participants thought about translation, and in what they did differently, dialogue-based approaches, such as STIR, offer a practical mechanism through which interdisciplinary science can become more societally responsive—not only by combining perspectives, but by reshaping how problems are framed and addressed at the individual level.

### 4.2. Practical implications

The findings carry several practical implications for health policymaking and implementation, capacity-building efforts, and collaborative research governance. First, our findings show that reflexive dialogue can complement system-level initiatives like evidence-informed policymaking (Lavis et al., 2009) and integrated knowledge translation (Kothari & Wathen, 2013) at the micro-level, by shaping how researchers engage with translation challenges in practice. Participants demonstrated enhanced anticipatory capacity by identifying potential social concerns and dealing with them with greater understanding, thus reframing outputs and research priorities.

Health policymaking and implementation processes benefit when researchers are better equipped to anticipate and align with the needs, values, and constraints of society and policymakers. Consistent with implementation science, effective evidence uptake depends not only on robust findings but also on researchers' engagement with social and contextual factors (Wensing & Grol, 2019). Reflexive practices—like those facilitated by STIR—equip scientists to consider these contextual dimensions more thoughtfully, thus improving their capacity to communicate in ways that resonate with diverse stakeholders and communities, ultimately supporting the systematic translation of evidence into practice and contributing to the broader goals of health system improvement. Our findings illustrate that STIR participants developed a more nuanced understanding of how their work may be interpreted, contested, or operationalized in social and policy contexts. For example, researchers began to recognize and consider the implications of their work on different groups, and responded with greater empathy towards society's concerns. This reflects a broader understanding that while scientists are often viewed as key sources of knowledge, that knowledge only benefits society when it is grounded in public trust—something that is built through genuine empathy for public needs and concerns (Peck, 2021). Consistent with implementation science frameworks emphasizing “context” (Nilsen & Bernhardsson, 2019), our study suggests that individual reflexivity can support more nuanced, practice-oriented engagement with contextual determinants.

Second, these findings have implications for the design of capacity-building programmes. Current initiatives aimed at improving researcher engagement with societal issues tend to emphasize technical skills (e.g., policy communication or stakeholder mapping) but often neglect the cultivation of epistemic humility and ethical sensitivity. The STIR process, by contrast, supports researchers in articulating and questioning their assumptions, contributing to “anticipatory governance” (Barben et al., 2008). In adapting STIR to a health-crisis context, it explicitly foregrounded urgency in research outcomes, population-level consequences and ethical sensitivity. These adaptations distinguish the present study from earlier STIR applications in RRI, such as Poznic & Fisher (2021) and Smolka & Fisher (2024), while aligning with prior work that has explored interdisciplinary collaboration in the health context (Crocker et al., 2018; Domecq et al., 2014; Kothari & Wathen, 2013).

Finally, since interdisciplinary collaboration is often challenged by differences in language, values, and epistemological assumptions, structured mechanisms for mutual learning are essential. Successful integration requires mutual questioning of disciplinary perspectives, awareness of institutional contexts, and a continuous flow of knowledge between fields (Kivits et al., 2019). By offering a structured, iterative approach to surfacing and negotiating these differences, STIR acts as a boundary object that enables meaningful collaboration without demanding full disciplinary convergence. This has implications for funders and institutions seeking to support interdisciplinary responses to complex health challenges. As suggested by Redman et al. (2021), success in these contexts depends not only on assembling diverse teams but also on cultivating shared reflexive practices that sustain collaborative engagement over time. Especially in high-pressure situations like pandemics, having such frameworks in place can improve the coherence and responsiveness of health research efforts.

Overall, STIR operationalizes midstream modulation in health research by offering observable changes in how researchers approach translation, collaboration, and public engagement. These findings suggest that reflexivity, when cultivated through structured dialogue, can play a critical role in making health research more aligned with real-world concerns and better integrated into actionable policy processes.

### 4.3. Limitations

While STIR offers a practical mechanism for fostering more societally responsive interdisciplinary science, several limitations are important to acknowledge. First, the high level of engagement and commitment needed by all sides during the process can discourage participation, since many researchers (especially in their early career phase) already deal with many challenges in time management, project deadlines and responsibilities. Second, as the STIR timeframe of ca. 12 weeks is restricted, more long-term changes and impact cannot be examined or measured, unless diligently followed up. Therefore, if the STIR exercise encourages reflection beyond this specific time, it remains hidden. Lastly, the difficulties in navigating emotional and institutional dynamics that influence collaborative reflection are a relevant constraint. This also includes potential desirability bias by the STIR researcher themselves, wherein they choose and make decisions on directing and asking questions where they see a potential for reflection. This requires experience and high levels of consideration on the side of the STIR researcher in order to remain objective towards possibilities of limited reflection on the part of the STIRed researcher. While the participant is asked about specific changes from the previous STIR session and the causes of it in the current session, it can be difficult to neatly separate professional evolution (even during a 12-week period) from the STIR-driven modulation when interpreting the results.

Mitigation of these restrictions is possible to a certain degree. Awareness of them is a first step that can help adapt certain steps and aspects during the STIR and to the unique demands of public health research and practice. Yet, the core aim of STIR makes it an intense exercise for all sides, since it cannot be reduced to reflection and responsiveness as a “box to click”. It is important that STIR is integrated and considered from the very beginning of the proposal and project phases, which can ensure targeted uses and possible analysis beyond the exact time frame. Overall, methods such as STIR are important to explore possibilities to enable and extend reflexivity. Therefore, experimenting with their implementation and learning from this remains an important task.

## 5. Conclusion

Empirically, this study shows that interdisciplinary collaboration, in this case using STIR, fosters changes in awareness and attitudes towards societal considerations, sometimes leading to concrete behavioural changes. These changes demonstrate how reflexivity is enacted at the level of individual early-career researchers. While institutional approaches to knowledge translation and evidence-informed policymaking remain essential, our findings suggest that supporting micro-level reflexive practices—particularly through structured interdisciplinary methods like STIR—can significantly enhance the translation of research into actionable public policy. Specifically, the STIR sessions revealed that by reflecting on assumptions, or recognizing downstream implications, the participants altered their research priorities and the value they gave to science communication.

In health crisis contexts, such as our case of the COVID-19 virus deactivating technologies, rapid and socially attuned decision-making is vital. The STIR interactions fostered these capacities—such as empathy towards society's fears, anticipation of societal concerns, and reframing scientific assumptions—enabling scientists to integrate these considerations midstream within the research process. Taken together, these findings contribute empirical evidence that reflexivity operates not as an abstract ethical ideal, but as a set of situated, interactional practices that can be deliberately supported through structured methods like STIR, for reflexivity and responsible innovation in health research. In the end, as Dr. Covello, director of the Centre for Risk Communication in New York, puts it, “People don't care what you know, they want to know that you care”. Reflexive practices like STIR move us closer to that kind of science—thoughtful, responsive, and deeply human.

## Data Availability

All the data collected, used, and/or analyzed for the current study, including voice recordings and transcripts, are available from the corresponding author upon reasonable request.

## Endnotes

^a^
For this study early career researchers were defined as individuals conducting their PhD or early postdoctoral research (their first postdoctoral position) at the time of the STIR. The CORAERO project had a dedicated PhD programme with annual PhD schools, which made this group accessible.

## List of abbreviations

COVID-19: Coronavirus Disease 2019
EBP: Evidence-Based Policymaking
EIP: Evidence-Informed Policymaking
IKT: Integrated Knowledge Translation
KT: Knowledge Translation
P[1/2/3/4]: participant [1/2/3/4]
RRI: Responsible Research and Innovation
STIR: Socio-Technical Integration Research
